# Handbook of Diseases of the Ear

**Published:** 1892-03

**Authors:** 


*Handbook of Diseases of the Ear.* By URBAN PRITCHARD, M.D. (Edin.), F.R.C.S. (Eng.). Second Edition. Pp. xvi., 238. London: H. K. Lewis. 1891.

We had occasion to notice favourably the first edition of this work, and the present issue follows the usual custom of having much new matter added, so that the size of the book is somewhat increased. The new material includes the important complications and sequelæ of middle-ear suppuration, including mastoiditis and cerebral abscess; it also includes a chapter on naso-pharyngeal disease. The whole is brought well up to date, and forms a valuable handbook for the student or practitioner.

